# Adult Ileocolic Intussusception Secondary to an Inflammatory Fibroid Polyp of the Terminal Ileum Presenting as Acute Small Bowel Obstruction: A Case Report

**DOI:** 10.7759/cureus.109891

**Published:** 2026-05-29

**Authors:** Sergio Heinar Rodríguez Sosa, Jesús Armando Ruvalcaba Velazquez, Maritza Aime Bello Olivera, Luis Fernando Rodríguez Ortiz, José Francisco Loyo Rodríguez

**Affiliations:** 1 General Surgery, Clinica Hospital Instituto de Seguridad y Servicios Sociales de los Trabajadores del Estado (ISSSTE), Mérida, MEX; 2 General Surgery, Instituto Politécnico Nacional, Mexico City, MEX; 3 General Surgery, Universidad Nacional Autónoma de México, Mexico City, MEX; 4 General Surgery, Universidad Marista de Mérida, Mérida, MEX

**Keywords:** exploratory laparotomy, inflammatory fibroid polyp, intussusception, small bowel obstruction, terminal ileum

## Abstract

Intussusception in adults is an uncommon cause of intestinal obstruction and is usually associated with an underlying structural lesion, unlike pediatric cases. In most adult patients, a pathological lead point is identified, frequently neoplastic in origin, making surgical management the standard treatment. We present the case of a 66-year-old man with a history of type 2 diabetes mellitus who presented with colicky abdominal pain, vomiting, and hematochezia. Physical examination revealed abdominal distension, tympany, diffuse tenderness, rebound tenderness, and hyperactive bowel sounds. Computed tomography demonstrated dilated small bowel loops with a transition zone and findings suggestive of ileocolic intussusception causing mechanical bowel obstruction. An exploratory laparotomy was performed, revealing ileocolic intussusception associated with two lesions in the terminal ileum located 15 and 25 cm from the ileocecal valve. Segmental resection of 30 cm of the terminal ileum with distal closure, terminal ileostomy, and incidental appendectomy was carried out. Histopathological examination reported an inflammatory fibroid polyp measuring 4 × 2.3 cm associated with ulcerated ischemic ileitis, without compromise of surgical margins. Adult intussusception should be considered secondary to an organic lesion until proven otherwise. Prompt diagnosis with computed tomography and early surgical intervention are essential to prevent ischemic complications. Although rare, inflammatory fibroid polyps may act as a pathological lead point for intussusception and bowel obstruction.

## Introduction

Adult intussusception is an uncommon clinical entity, accounting for approximately 1% of all cases of bowel obstruction and less than 5% of all intussusceptions [1-3]. Unlike pediatric cases, where the etiology is usually idiopathic, most adult cases are associated with a definable structural lesion that serves as a pathological lead point [2,3]. Both benign and malignant neoplasms are among the most frequent underlying causes, making prompt diagnosis and surgical management essential [1,4]. Distinguishing benign lesions from malignant etiologies is particularly important in adult patients because it directly influences surgical decision-making and overall prognosis.

Inflammatory fibroid polyp is a rare benign mesenchymal lesion of the gastrointestinal tract that most commonly arises in the gastric antrum and small bowel [5,6]. Histologically, these lesions are characterized by spindle-cell proliferation with eosinophilic inflammatory infiltrates involving the submucosa [5,6]. Although generally asymptomatic, lesions involving the ileum may act as a pathological lead point for intussusception and subsequent bowel obstruction [4,5]. Due to its low incidence, reports describing ileocolic intussusception secondary to inflammatory fibroid polyps in adults remain limited in the literature [4,5].

Clinical presentation in adults is often nonspecific and may include intermittent abdominal pain, nausea, vomiting, gastrointestinal bleeding, or signs of mechanical bowel obstruction, frequently leading to delayed diagnosis [2,7,8]. Computed tomography has become the diagnostic modality of choice because of its high sensitivity in identifying intussusception, transition zones, bowel compromise, mesenteric vessel invagination, and associated lesions [7,9,10].

We present the case of an adult patient with ileocolic intussusception secondary to an inflammatory fibroid polyp of the terminal ileum presenting as acute mechanical bowel obstruction requiring emergency surgical management.

## Case presentation

A 66-year-old man with a medical history significant for type 2 diabetes mellitus and unspecified prior foot surgery presented to the emergency department with a one-day history of colicky abdominal pain, multiple episodes of vomiting, and hematochezia. He denied tobacco use, reported chronic alcohol consumption, and had no known drug allergies.

On physical examination, the patient was conscious and oriented, with pallor in the skin and mucous membranes. Abdominal examination revealed distension, tympany, diffuse tenderness to palpation, rebound tenderness, muscular guarding, and hyperactive bowel sounds suggestive of mechanical bowel obstruction. Cardiopulmonary examination showed no significant abnormalities.

Initial laboratory studies showed mild anemia and renal dysfunction, as shown in Table 1.

**Table 1:** Laboratory findings on admission

Parameter	Result	Units	Reference range
White blood cell count	5.13	×10³/mm³	4.5-10
Hemoglobin	12.2	g/dl	10-15
Hematocrit	35.2	%	30-46
Platelet count	313	×10³/mm³	150-400
Glucose	133	mg/dL	70-105
Blood urea nitrogen	46.7	mg/dL	07-25
Urea	100	mg/dL	17.10-42.80
Creatinine	2.52	mg/dL	0.60-1.20
Prothrombin time	13	seconds	11-15.1
International normalized ratio	1	-	0.87-1.2
Activated partial thromboplastin time	32	seconds	31.8-43.7
Sodium	138	mmol/L	136-145
Potassium	3.5	mmol/L	3.5-5.10
Chloride	100	mmol/L	98-107

Contrast-enhanced abdominal computed tomography (Figure 1) demonstrated marked gastric and small bowel dilatation with a transition zone at the distal ileum. Findings were consistent with ileocolic intussusception causing mechanical small bowel obstruction, with associated bowel wall thickening and mesenteric vessel invagination suggestive of a pathological lead point.

**Figure 1:**
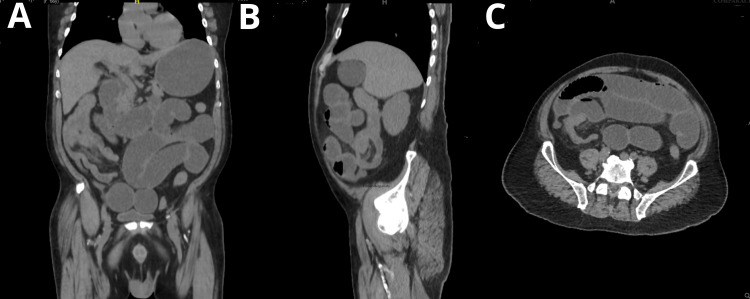
Contrast-enhanced abdominal computed tomography demonstrating ileocolic intussusception causing small bowel obstruction (A) Coronal section showing marked dilatation of small bowel loops with bowel wall thickening and ileocolic intussusception. (B) Sagittal section demonstrating telescoping of the distal ileum into the colon associated with proximal intestinal dilatation. (C) Axial section revealing the characteristic target appearance of intussusception with dilated fluid-filled bowel loops and mesenteric vessel invagination.

Given the suspicion of mechanical bowel obstruction secondary to an underlying structural lesion, the patient underwent emergency exploratory laparotomy. Intraoperatively, approximately 100 mL of reactive peritoneal fluid was identified, along with ileocolic intussusception involving the terminal ileum (Figure 2). Two abnormal areas were identified in the terminal ileum, approximately 15 and 25 cm from the ileocecal valve. One corresponded to the inflammatory fibroid polyp identified on histopathology, while the second represented ischemic and inflammatory bowel wall thickening associated with ulcerated ischemic ileitis (Figure 3).

**Figure 2:**
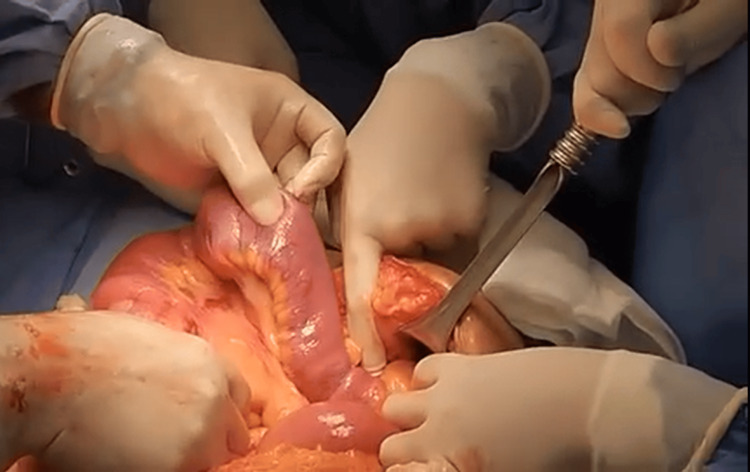
Intraoperative image demonstrating ileocolic intussusception involving the terminal ileum

**Figure 3:**
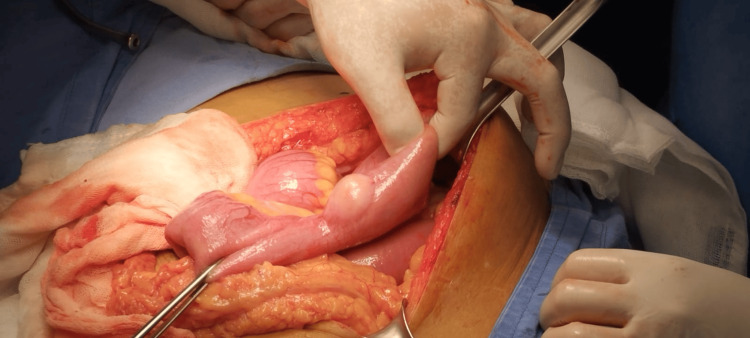
Intraoperative image of the inflammatory fibroid polyp and ischemic bowel involvement of the terminal ileum

Segmental resection of 30 cm of the terminal ileum was performed with distal closure in Hartmann fashion and terminal ileostomy maturation using the Brooke technique. Primary anastomosis was avoided because of bowel obstruction, ischemic changes, reactive peritoneal fluid, and increased risk of anastomotic complications in the emergency setting. An incidental appendectomy was also performed because of reactive inflammatory changes involving the appendix.

The postoperative course was favorable, with adequate oral tolerance, functional ileostomy output, and no postoperative complications. Histopathological examination revealed an inflammatory fibroid polyp measuring 4 × 2.3 cm involving the submucosa and muscularis propria without compromise of surgical margins (Figures 4-5). Microscopic evaluation demonstrated spindle-cell proliferation with eosinophilic inflammatory infiltrates involving the submucosa and deeper layers of the bowel wall, findings consistent with an inflammatory fibroid polyp. Immunohistochemical studies were not available in this case. An associated ulcerated ischemic ileitis was also identified. Mesenteric lymph nodes showed no pathological alterations, and the appendix demonstrated no significant histological abnormalities.

**Figure 4:**
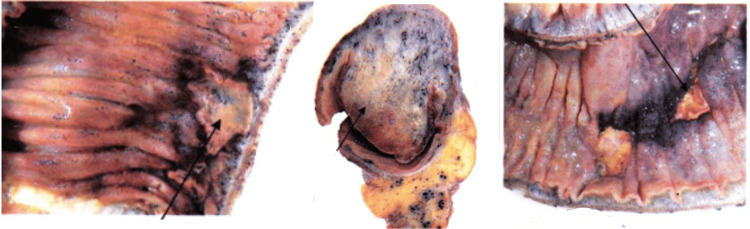
Macroscopic specimen demonstrating inflammatory fibroid polyp of the terminal ileum associated with ulcerated ischemic lesions

**Figure 5:**
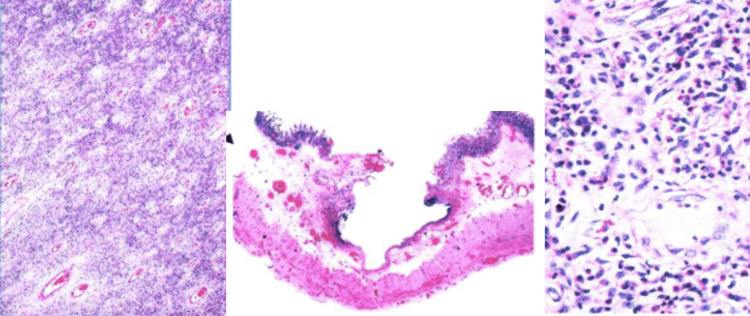
Histopathological examination showing spindle-cell proliferation with eosinophilic inflammatory infiltrates involving the submucosa, consistent with inflammatory fibroid polyp

## Discussion

Adult intussusception differs significantly from pediatric intussusception regarding etiology, clinical presentation, and management [1-3]. While pediatric cases are usually idiopathic, adult intussusception is associated with an identifiable pathological lead point in up to 90% of patients [2,3]. In the small bowel, benign lesions are more frequently implicated, whereas colonic intussusception carries a substantially higher probability of malignancy. Because of this strong association with structural pathology, surgical resection remains the standard treatment in adult patients [1,8].

Clinical presentation in adults is frequently nonspecific and may be acute, intermittent, or chronic, making preoperative diagnosis challenging [2,8]. Patients commonly present with abdominal pain, nausea, vomiting, gastrointestinal bleeding, or signs of bowel obstruction [1-3]. In the present case, the patient developed acute obstructive symptoms associated with hematochezia and peritoneal irritation, findings suggestive of bowel compromise requiring urgent surgical intervention.

Computed tomography is considered the gold-standard imaging modality because it identifies characteristic target lesions, bowel compromise, mesenteric vessel invagination, transition zones, and possible ischemia [7,9,10]. In this patient, computed tomography confirmed the diagnosis of ileocolic intussusception. It demonstrated marked small bowel dilatation with mesenteric vessel invagination, findings highly suggestive of a pathological lead point and mechanical obstruction.

Inflammatory fibroid polyps are rare benign mesenchymal lesions of the gastrointestinal tract and are considered uncommon causes of adult intussusception [4-6]. These lesions are most commonly located in the gastric antrum, followed by the ileum, where they may become symptomatic due to luminal obstruction or serve as a lead point for intussusception [4,5]. Histologically, they are characterized by spindle-cell proliferation with eosinophilic inflammatory infiltrates involving the submucosa and deeper layers of the bowel wall [5,6]. Differential diagnosis includes other spindle-cell mesenchymal lesions such as gastrointestinal stromal tumor, inflammatory myofibroblastic tumor, and schwannoma. Immunohistochemical studies may be useful for definitive differentiation; however, these studies were not available in the present case. Despite their benign nature, preoperative distinction from malignant neoplasms remains difficult, particularly in emergency surgical settings [4].

The surgical approach to adult intussusception remains controversial, particularly regarding prior reduction versus primary resection. Most authors recommend intestinal resection without prior reduction when malignancy, bowel ischemia, or perforation is suspected because of the potential risk of perforation, tumor dissemination, and anastomotic complications [8,9]. In our patient, the presence of bowel obstruction, ischemic bowel changes, reactive peritoneal fluid, and a suspected structural lesion justified immediate intestinal resection without extensive manipulation. Primary anastomosis was avoided because of the increased risk of postoperative complications in the emergency setting, favoring terminal ileostomy as a staged surgical approach.

This case highlights the importance of maintaining a high index of suspicion for adult intussusception in patients presenting with acute bowel obstruction and atypical gastrointestinal bleeding [1,2]. Although inflammatory fibroid polyps are rare, they should be considered in the differential diagnosis of pathological lead points in the small bowel [4-6]. Early recognition, appropriate imaging evaluation, histopathological confirmation, and prompt surgical management are essential for preventing ischemic and septic complications and achieving favorable clinical outcomes [1,3].

## Conclusions

Adult intussusception is a rare but clinically significant cause of mechanical bowel obstruction and should raise suspicion for an underlying structural lesion. Inflammatory fibroid polyps, although uncommon, may serve as a pathological lead point in the small bowel and can mimic malignant neoplasms in emergency settings. Computed tomography plays a critical role in preoperative diagnosis by identifying bowel obstruction, intussusception, and associated pathological findings. Histopathological evaluation remains essential for definitive diagnosis and differentiation from other mesenchymal lesions. Early surgical intervention is fundamental to preventing ischemic complications and achieving favorable clinical outcomes.
